# Intrabasin Variability of East Pacific Tropical Cyclones During ENSO Regulated by Central American Gap Winds

**DOI:** 10.1038/s41598-017-01962-3

**Published:** 2017-05-10

**Authors:** Dan Fu, Ping Chang, Christina M. Patricola-DiRosario

**Affiliations:** 1 Physical Oceanography Laboratory/CIMST, Ocean University of China and Qingdao National Laboratory for Marine Science and Technology, Qingdao, 266100 China; 2 Department of Oceanography, Texas A&M University, College Station, Texas 77843 USA; 3 Climate and Ecosystem Sciences Division, Lawrence Berkeley National Laboratory, Berkeley, California, 94720 USA; 4 Department of Atmospheric Sciences, Texas A&M University, College Station, Texas 77843 USA

**Keywords:** Atmospheric dynamics, Physical oceanography, Atmospheric dynamics

## Abstract

Hurricane Patricia in 2015 was the strongest Pacific hurricane to make landfall in Mexico. Although Patricia fortuitously spared major cities, it reminded us of the threat tropical cyclones (TCs) pose in the eastern North Pacific (ENP) and the importance of improving our understanding and prediction of ENP TCs. Patricia’s intensity and the active 2015 ENP hurricane season have been partially attributed to the strong El Niño in 2015, however there is still a lack of fundamental understanding of the relationship between El Niño-Southern Oscillation (ENSO) and ENP TCs. Here, we demonstrate that ENSO drives intrabasin variability of ENP TCs, with enhanced (reduced) TC frequency in the western portion of the ENP during El Niño (La Niña), but reduced (enhanced) TC frequency in the eastern nearshore area, where landfalling TCs preferentially form. This intrabasin difference is primarily driven by the Central American Gap Winds (CAGW), which intensify (weaken) during El Niño (La Niña), producing low-level anticyclonic (cyclonic) relative vorticity anomalies and thus an unfavorable (favorable) environment for TC genesis. These findings shed new light on the dynamics linking ENP TC activity to ENSO, and highlight the importance of improving CAGW representation in models to make skillful seasonal forecasts of ENP TCs.

## Introduction

The ENP basin (0–30°N, 140°W to the North American coast)^1^ is the most active TC genesis region on the globe, in terms of genesis per unit area^2^. TC landfalls on the Pacific coast of Central America and Mexico are relatively infrequent due to the tendency for westward/northwestward TC trajectories. However, those TCs that do make landfall can bring catastrophic flooding, especially when TC-associated winds and moisture interact with mountainous orography^3–5^. For example, significant coastal flooding was triggered by Hurricanes Pauline in 1997 and Patricia in 2015. The latter is the strongest hurricane on record for the Western Hemisphere and inflicted an estimated $460 million USD damage across Mexico and Southern Texas^6, 7^. Observed historical TC tracks in the ENP indicate that all landfalling TCs formed east of 116°W (Supplementary Fig. 1). Therefore, gaining a better understanding of the factors controlling TC variability in this Eastern Development Region (EDR; term first introduced in a previous study^8^) of the ENP has both outstanding scientific motivation and profound socioeconomic implications.

El Niño-Southern Oscillation (ENSO) is the most dominant climate mode on interannual timescales^9^, and is a critical predictor in seasonal TC forecasts^10, 11^ During El Niño, vertical wind shear over the ENP is reduced in response to anomalously warm eastern-central Equatorial Pacific sea surface temperature (SST), which tends to favor above-normal ENP TC activity^12^. Anomalous eastern Pacific ocean heat content during El Niño can also affect TC intensity^13^. However, whether there is a statistically robust relationship between ENSO and observed TC activity over the ENP has been called into question^1, 14^. By dividing the ENP into Western Development Region (WDR) and EDR, recent studies^8, 14–16^ found that only TCs that originated in the WDR are robustly correlated with ENSO (*p*-value < 0.05), with more frequent and intense TCs observed during El Niño than La Niña. Locations of TC genesis and dissipation within the ENP are also characterized by a westward shift during El Niño compared to neutral ENSO and La Niña^17^. Such a TC response in the WDR has been attributed to changes in SST and mid-tropospheric relative humidity during ENSO^15, 18^. However, ENSO’s impact on TCs in the EDR, which have a much higher probability to make landfall along the Pacific coast of Central America, has not been thoroughly investigated. There is currently no consensus on whether and how ENSO can exert an influence on TCs in the EDR.

Many previous studies on ENSO’s impact on ENP TCs used area-integrated measures, such as TC counts, TC days and the power dissipation index (PDI)^19^, to relate ENP TC variability with ENSO indices^14–16^. However, few studies explored the influence of ENSO on the spatial distribution of TCs within the ENP, and the associated mechanism. As will be demonstrated below, a distinctive signature of ENSO’s impact on ENP TCs is an east-west spatial variation in TC activity. Such a feature cannot be understood using area-integrated analyses, which average out the opposite changes of TC activity in different parts of the basin. To address this issue, we carried out analyses focusing on ENSO’s impact on the spatial distribution of TCs within the ENP. We note that the boundary separating the EDR and WDR in our study is set at 116°W, following a previous study^8^. However, our analyses do not include Central Pacific TCs between 180° and 140°W, because the focus of this study is on the eastern part of the ENP near the coastline where mountain influences are potentially greater. Thus our WDR extends only to 140°W, rather than to the dateline^8^.

## Results

We first made a composite of TC genesis location and track density for all El Niño and La Niña events during the period from 1970 to 2015 and compared the results to the climatological genesis location and track density for this period. 13 El Niño and 11 La Niña hurricane seasons were identified based on the 75th and the 25th percentiles of the Niño3.4 index (Supplementary Fig. 2; details in Methods).

**Figure 1:**
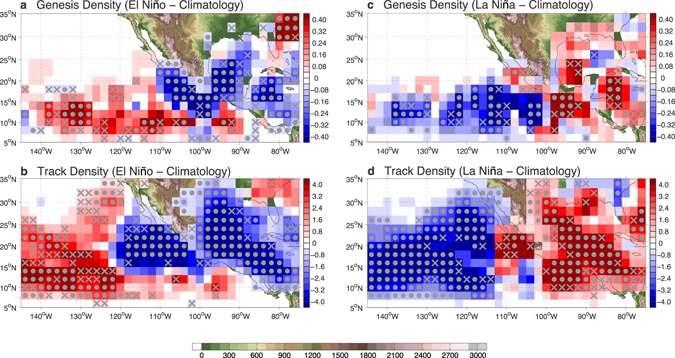
ENP TC variability during El Niño and La Niña. The El Niño hurricane seasons composite minus climatology difference in (**a**) TC genesis density (unit: TCs per 10 seasons), and (**b**) TC track density (unit: TCs per 10 seasons). (**c**), (**d**) are same as (**a**), (**b**) but for the difference from the La Niña hurricane seasons composite minus climatology. Gray dots (crosses) denote statistical confidence at the 95% (90%) level. Statistical significance test is performed based on the two-sample t-test. Land topography (unit: m) is shaded green-brown. The maps were generated using M_Map V1.4 package for Matlab (http://www.eos.ubc.ca/~rich/map.html).

Figure 1 shows the difference in TC genesis and track density (defined in Methods) averaged over the 13 El Niño and 11 La Niña events relative to the 46-year (1970–2015) climatology. It is evident that, despite the marked increase of TC activity in the WDR that is consistent with previous studies^15, 16^, El Niño suppresses TCs in the EDR, as in the Caribbean and Gulf of Mexico. As a result, both TC genesis (Fig. 1a) and track density (Fig. 1b) in the ENP exhibit a westward shift during El Niño, with a prevailing decrease near the Pacific coast of Central America and the western side of the Sierra Madre Mountains. We examined the robustness of this westward shift pattern by performing a Student’s t-test and a bootstrap statistical test (Supplementary Fig. 3; details in Methods), as well as by performing a regression analysis where both TC genesis and track density were regressed onto the hurricane season mean Niño3.4 index (Supplementary Fig. 3). The fact that the response pattern survives this statistical scrutiny gives us increased confidence that El Niño can, in a statistical sense, cause a decrease (increase) in TC activity in the EDR (WDR) of the ENP.

Similar spatial variability of ENP TCs, but opposite in sign, occurs during La Niña, with an expected decrease of TC activity in the WDR and an increase in the EDR (Fig. 1c,d). These findings point to an east-west spatial pattern in the ENP TC response to ENSO, which we refer to as intrabasin TC variability. The average TC frequency change caused by the intrabasin variability in the nearshore area is comparable to the well-known ENSO-induced TC change in the North Atlantic. Within 550 km off the west coast of Central America, the distance within which TC centers can generate observable rainfall at coastal stations^5^, the TC frequency decreases by ~22.4% (increases by ~16.2%) during El Niño (La Niña). These changes are similar in magnitude to those observed in the Caribbean and Atlantic during ENSO^10^. This ENSO-related ENP TC activity change in the nearshore area is also consistent with a previous study^20^.

We next examine whether this intrabasin TC variability is consistent with our current understanding of the dynamic processes controlling TC variations. To do so, we evaluate the genesis potential index (GPI^21^; details in Methods) using the National Centers for Environmental Prediction (NCEP) Climate Forecast System Reanalysis (CFSR^22^, 1979–2010) and its extension, the Climate Forecast System version 2 (CFSv2^23^; 2011–2015; details in Methods). We chose CFSR and CFSv2 because they produce a more realistic representation of fine-scale atmospheric circulation features over the EDR^24^ than other reanalysis products. However, owing to the shorter record length of CFSR and CFSv2 data, only 11 El Niño and 7 La Niña hurricane seasons were identified during the 1979–2015 period. But, the TC activity response to El Niño and La Niña is insensitive to changes in the record length (Supplementary Fig. 4; details in Methods), providing justification for using the shorter period composite in the following diagnostic analyses.

**Figure 2:**
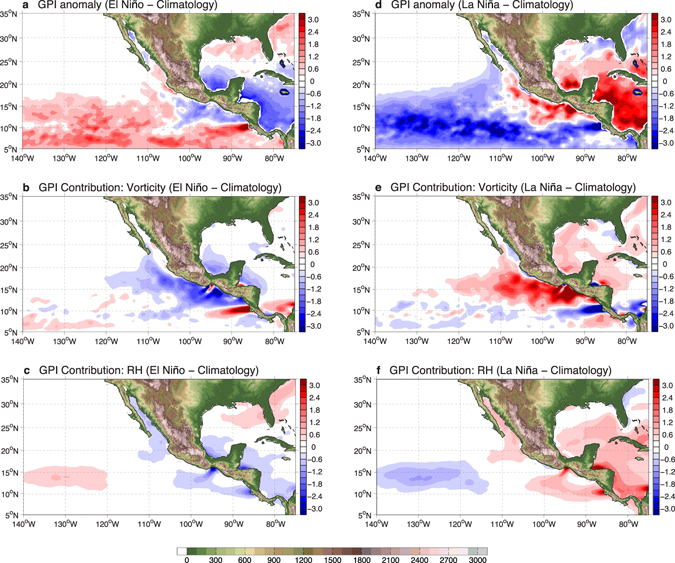
The impact of El Niño and La Niña on environmental favorability for TC genesis. (**a**) The El Niño hurricane seasons composite minus climatology difference in genesis potential index (GPI, unitless) and difference of GPI (unitless) calculated by varying just (**b**) vorticity, and (**c**) relative humidity, while setting the other three terms to values of the climatology. (**d**), (**e**), and (**f**) are similar, but for the GPI anomaly from the La Niña hurricane seasons composite minus climatology. Positive indicates environmental conditions are more favorable for TC genesis, and vice versa. Land topography (unit: m) is shaded green-brown. The maps were generated using M_Map V1.4 package for Matlab (http://www.eos.ubc.ca/~rich/map.html).

GPI is widely used to examine environmental favorability for TC genesis, and the seasonal cycle of GPI averaged over the ENP main development region (MDR; 8°−20°N, 140°W to the North American coast; see gray box in Supplementary Fig. 5c) indeed corresponds well with the observed number of TCs (Supplementary Fig. 5a). The correlation between MDR-averaged hurricane season mean GPI and observed ENP TC frequency during 1979–2015 has a value of 0.59 (*p*-value < 0.01 based on the Student’s t-test) (Supplementary Fig. 5b), indicating that GPI describes the basin-wide ENP TC variability well^25, 26^. Consistent with the observed intrabasin TC variability shown in Fig. 1, GPI anomalies during El Niño display an east-west pattern with increased GPI values in the WDR and decreased GPI values in much of the EDR (Fig. 2a). During La Niña, GPI anomalies are also characterized a similar spatial pattern, but with the opposite sign (Fig. 2d).

To identify the dominant atmospheric processes that drive intrabasin TC variability, we further evaluated the relative importance of various atmospheric parameters that determine environmental favorability for TCs (details in Methods). Over much of the ENP, the El Niño-induced changes in vertical wind shear and hurricane maximum potential intensity provide more favorable conditions for tropical cyclogenesis (Supplementary Fig. 6), consistent with a previous study^25^. However, in the EDR, the changes in vorticity and relative humidity create less favorable conditions for TC genesis (Fig. 2b,c). The negative GPI anomaly due to changes in vorticity is particularly important, and dominates over that due to humidity changes near the coastal area (Fig. 2b,c). Combined, these negative GPI anomalies overpower the positive GPI anomalies associated with changes in vertical wind shear and hurricane maximum potential intensity, resulting in a net negative GPI anomaly in the near coastal region (Fig. 2a). As expected, GPI anomalies during La Niña show nearly the same patterns, but opposite in sign, and the positive GPI anomalies near the Pacific coast are again attributed to the vorticity and relative humidity changes (Fig. 2e,f).

Of particular note is that both the low-level vorticity-induced and mid-tropospheric moisture-induced negative (positive) GPI anomalies during El Niño (La Niña) show a well-defined structure extending westward from the Sierra Madre topographic gap areas, especially near the Isthmus of Tehuantepec (hereafter, TT; Supplementary Fig. 7) and Gulf of Papagayo (hereafter, PP; Supplementary Fig. 7). This prompted us to probe further into the connection between the topographically-locked features and the ENP intrabasin TC variability during El Niño (La Niña), particularly the TC suppression (enhancement) in the EDR.

The Sierra Madre blocks large-scale, low-level flow from North America and the Caribbean towards the ENP. There are three major narrow topographic gaps at TT, PP and Panama (PN), and the depth of these gaps can approach ~1000 meters vertically (Supplementary Fig. 7). During boreal winter (November through March), intermittent winter storms from the Gulf of Mexico and more steady trade winds force strong wind jets penetrating through these narrow gaps and into the ENP, generating the Central American Gap Winds (CAGW)^27–29^. Although the CAGW at TT and PP are strongest during boreal winter^28, 29^, they are also characterized by a secondary peak during July-August caused by the westward intensification of the Azores-Bermuda high pressure system^29^.

**Figure 3:**
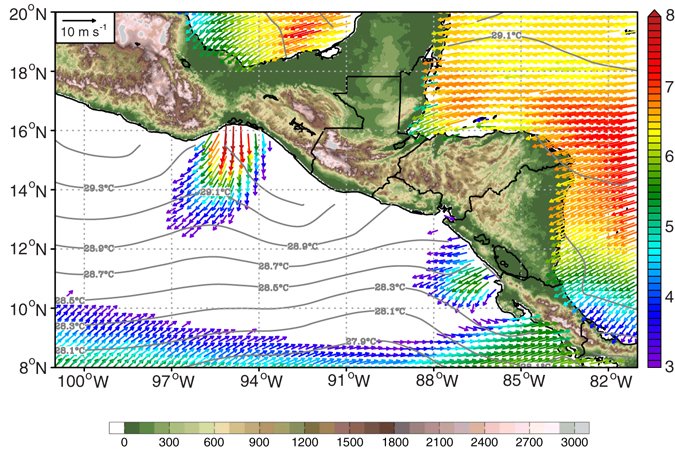
Climatological ocean surface wind and SST. Climatological hurricane season averaged ocean surface wind (vectors, unit: m s^−1^) is derived from QuikSCAT V4 wind product. Colors of vectors indicate wind speeds as shown in the right colorbar, only plotting the region where the wind speed is higher than 3 m s^−1^. Climatological hurricane season averaged SST (contours, unit: °C) is plotted at intervals of 0.2 °C. Land topography (unit: m) is shaded green-brown. The map was generated using GrADS V2.1.0 (http://cola.gmu.edu/grads/).

Figure 3 shows the 1999–2009 hurricane season mean ocean surface winds derived from National Aeronautics and Space Administration’s (NASA’s) Quick Scatterometer (QuikSCAT) Version-4 (V4) wind product^30^ (details in Methods), along with the corresponding SST. Both CAGW at TT and PP are clearly visible in the climatology, but the northerly TT jet is stronger and more effectively cools local SSTs. The CAGW at TT follows anticyclonic arcs downwind from the coast. In contrast, the CAGW at PP is more zonal because of the orientation of topographic gaps and the influence of steady trade winds. ENSO modulates the strength of the CAGW^24, 31^, with intensified winds observed at both TT and PP during El Niño, due to an increased frequency of cold frontal intrusions^31^ and a southward shift of the Intertropical Convergence Zone (ITCZ)^9, 24^.

**Figure 4:**
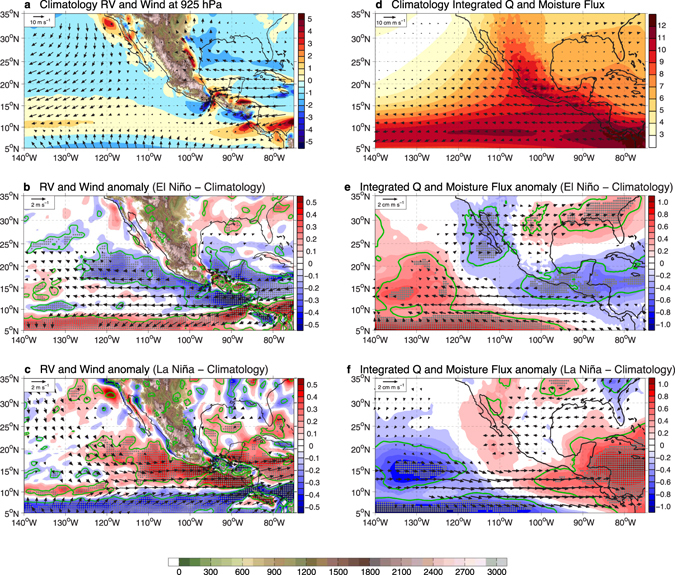
The impact of El Niño and La Niña on low-level relative vorticity and mid-level moisture. (**a**) Climatological, (**b**) the El Niño hurricane seasons and (**c**) the La Niña hurricane seasons composite minus climatology difference in relative vorticity (*rv*; shadings, unit: 10^−5^ s^−1^) and wind vectors (unit: m s^−1^) at 925 hPa. (**d**) Climatological, (**e**) the El Niño hurricane seasons and (**f**) the La Niña hurricane seasons composite minus climatology difference in 700 hPa through 500 hPa integrated specific humidity (*q*; shadings, unit: mm) and moisture flux (*q* ***V***; vectors, unit: cm m s^−1^). Gray dots (green contours) highlight the statistical confidence at the 95% (90%) level. All vector anomalies are plotted only where the confidence level is above 95%. Statistical significance test is performed based on the two-sample t-test. Land topography (unit: m) at atmospheric pressure level is shaded green-brown. The maps were generated using M_Map V1.4 package for Matlab (http://www.eos.ubc.ca/~rich/map.html).

Because of its narrow structure and strength, the CAGW plays a dominant role in determining low-level relative vorticity in the EDR, which is a key factor in influencing environmental favorability for tropical cyclogenesis. Figure 4a–c show the climatology, and the El Niño- and La Niña-induced anomalies of 925 hPa relative vorticity in the ENP, respectively, along with the 925 hPa wind vector. It is evident that the stronger horizontal wind shear associated with the CAGW at TT and PP generates two relative vorticity dipoles in the EDR (Fig. 4a). The negative pole near TT is particularly pronounced, because the anticyclonic curvature of the jet path after it leaves the Gulf of TT strengthens the existing anticyclonic relative vorticity.

During El Niño, the intensified CAGW at both PP and TT (Supplementary Fig. 8a) contributes to the anticyclonic (negative) relative vorticity anomalies north of 10°N, especially between TT and PP and along the western flank of the TT jet (Fig. 4b), resulting in a less favorable environment for tropical cyclogenesis (Fig. 2b) and TC suppression in the EDR^12, 32–35^. The southern flank of the PP jet, on the other hand, is characterized by a cyclonic (positive) relative vorticity anomaly. During La Niña, however, the reduced CAGW at TT and PP (Supplementary Fig. 8b) generates positive relative vorticity anomalies, which make the environment more favorable for TC genesis in the EDR (Figs 2e and 4c). These changes in the relative vorticity are remarkably consistent with observed negative (positive) genesis density and track density anomalies in the EDR during El Niño (La Niña). We thus conclude that the CAGW-induced low-level relative vorticity change during ENSO serves as a primary cause for the ENP TC intrabasin variability, particularly in the EDR.

A secondary but important factor contributing to TC variability in the EDR is the mid-tropospheric moisture change. Figure 4d–f show climatological 700 hPa – 500 hPa integrated specific humidity, overlaid by the integrated moisture flux (details in Methods), and the corresponding anomalies during El Niño and La Niña. At tropospheric mid-levels, above the extent of the CAGW, the integrated specific humidity over the EDR is reduced during El Niño (Fig. 4e) and increased during La Niña (Fig. 4f). The reduced (increased) mid-tropospheric moisture, during El Niño (La Niña) further contributes to the negative (positive) GPI anomaly and provides another source to suppress (enhance) TC activity in the EDR^15, 35, 36^. Further investigation is needed to understand the underlying dynamic mechanism responsible for the moisture changes in the region.

## Discussion

This study provides a new perspective on the impact of ENSO on ENP TC activity. It suggests that the previous view of increased (decreased) basin-wide ENP TC activity during El Niño (La Niña) caused by changes in vertical wind shear, SST, and mid-tropospheric relative humidity is incomplete. The ENP TC response to ENSO is characterized by intrabasin variability, with enhanced TC activity confined to only the WDR and reduced TC activity in the EDR during El Niño, and the opposite during La Niña. Furthermore, this study identifies a topographically locked feature – CAGW– as a primary player in regulating TC activity in the EDR through low-level relative vorticity changes. As all TCs that make landfall onto the Pacific coast of Central America and Mexico form in the EDR, the results presented here have important implications for improving seasonal forecast skill of ENP TCs and their impacts. It points to the necessity of properly resolving and representing the CAGW and its response to ENSO in TC forecast models.

We note that the overall reduction of TC activity over the EDR in response to El Niño does not exclude occurrences of individual extreme hurricanes in the region during El Niño. Indeed, Hurricane Patricia in 2015 and Hurricane Pauline in 1997, both among the strongest hurricanes to make landfall over Mexico, occurred during strong El Niño years. Individual hurricane genesis can be initiated by short-lived synoptic events that may not contribute significantly to the mean environment for TC genesis. Hurricane Patricia, for example, appeared to be initiated by a synoptic-scale CAGW strengthening event near TT that provided an injection of cyclonic relative vorticity on the cyclonic shear side and spurred the formation of the initial tropical depression^7^. These types of synoptic CAGW events are especially effective in triggering tropical cyclogenesis when they interact with the monsoon trough^37^. Understanding synoptic CAGW events as a mechanism of tropical cyclogenesis is an active research area, and more studies are clearly needed to elucidate the underlying dynamical processes and the distinction between their roles in synoptic versus seasonal and longer timescales.

## Methods

In this study, all composite and diagnostic analyses focus on the period from June 1^st^ through November 30^th^ (hereafter ‘hurricane season’), which accounts for more than 95% (792 out of 832) of the total number of TCs during 1970–2015. Note that our analysis period is slightly different from the ENP hurricane season defined by the National Hurricane Center (NHC), which runs from May 15^th^ through November 30^th^. Since the number of TCs during our research period covers more than 95% of total number, it is unlikely that the results are sensitive to this difference in hurricane season definition.

We use the hurricane season averaged Niño3.4 index derived from the detrended HadISST sea surface temperature dataset of the Met Office Hadley Centre^38^ to define ENSO events. The seasonal mean values above the 75th (below the 25th) percentile are chosen as El Niño (La Niña) hurricane seasons. During the period from 1970–2015, 13 El Niño hurricane seasons (1972, 1976, 1982, 1986, 1987, 1991, 1994, 1997, 2002, 2004, 2006, 2009, 2015) and 11 La Niña hurricane seasons (1970, 1971, 1973, 1975, 1988, 1998, 1999, 2000, 2007, 2010, 2011) are identified (Supplementary Fig. 2), which is in general agreement with previous studies^11, 16, 25^.

The World Meteorological Organization (WMO) subset of International Best Track Archive for Climate Stewardship (IBTrACS-WMO)^39^ dataset v03r09 is used to calculate genesis density and track density field. In order to better resolve spatial variability within the ENP basin, track density on a yearly basis is defined as the total number of 6-hourly TC central locations within each 2° (latitude) ×2° (longitude) square box during each hurricane season. To reduce noise and better capture principal anomaly patterns of TC locations, track density is then smoothed by averaging the surrounding eight points. Genesis density has a similar definition, except only the number of tropical cyclogenesis events (first record of TC that develops to at least a tropical depression) in each 2° × 2° square box are counted. Genesis density is also smoothed in the same way as track density.

In addition to the Student’s t-test, the bootstrap technique^40^ is also applied to determine the statistical significance level of both genesis and track density. The El Niño composite anomaly is constructed with 13 years randomly chosen from among a total of 46 years. This re-sample process is then repeated 10,000 times to obtain a 95% and 90% confidence interval. The same process is also applied for the La Niña composite anomaly, but by choosing 11 years out of 46-year climatology.

To reveal the underlying physical mechanism of intrabasin ENP TC variability, the National Centers for Environmental Prediction (NCEP) Climate Forecast System Reanalysis (CFSR^22^) and its extension, the NCEP Climate Forecast System version 2 (CFSv2)^23^, are used for TC environmental condition analyses. Both of these datasets are at a high spatial resolution of 0.5° × 0.5°. The CFSR was completed over a 32-year period from January 1979 to March 2011, and then the dataset was extended to the present as CFSv2. To better represent TC environmental conditions, we use 6-hourly rather than monthly mean reanalysis data. 11 El Niño (1982, 1986, 1987, 1991, 1994, 1997, 2002, 2004, 2006, 2009, 2015) and 7 La Niña (1988, 1998, 1999, 2000, 2007, 2010, 2011) hurricane seasons are identified during the entire 37-year period from 1979 to 2015 and are used to diagnose TC environmental condition changes during El Niño and La Niña events.

The environmental favorability for tropical cyclogenesis is assessed by analyzing the genesis potential index^21^ (GPI), which is defined as:

1 $${\rm{GPI}}={|{10}^{5}\eta |}^{3/2}{(\frac{H}{50})}^{3}{(\frac{{V}_{pot}}{70})}^{3}(1+0.1{V}_{shear})$$

where *η* is absolute vorticity at 850 hPa (s^−1^), *H* is relative humidity at 700 hPa (%), *V*_shear_ is the magnitude of vertical wind shear between 850 and 200 hPa (m s^−1^). *V*_pot_ is hurricane maximum potential intensity (PI^41–43^; m s^−1^), defined as:

2 $${V}_{pot}^{2}={C}_{P}({T}_{S}-{T}_{O})\frac{{T}_{S}}{{T}_{O}}\frac{{C}_{k}}{{C}_{D}}(\mathrm{ln}\,{\theta }_{S}^{\ast }-\,\mathrm{ln}\,{\theta }_{S})$$

where *T*_*S*_ is SST (K) and *T*_*o*_ is outflow air temperature at tropopause (K).*C*_*P*_ is heat capacity of air,*C*_*k*_ and *C*_*d*_ are the surface exchange coefficients of enthalpy and the drag coefficient (dimensionless), respectively. θ*_s_ and *θ*_*s*_ are surface saturation equivalent potential temperature and boundary layer equivalent potential temperature (K). The application of PI theory is discussed online (http://wind.mit.edu/~emanuel/holem/node3.html). FORTRAN and MATLAB subroutines are available online (ftp://texmex.mit.edu/pub/emanuel/TCMAX/). To estimate the primary contributions that lead to the ENSO-induced GPI anomalies, the GPI is also recalculated by interannually varying just one term while setting the other three terms to values of the long-term climatology^25, 44, 45^.

The ocean surface wind vector is derived from the National Aeronautics and Space Administration’s (NASA’s) Quick Scatterometer (QuikSCAT) Version-4 (V4) wind product^30^. This dataset is at a horizontal resolution of 0.25° × 0.25°, based on an improved Ku-band Geophysical Model Function (GMF) to optimize wind speed retrievals, especially in high wind speed areas. QuikSCAT ocean surface wind is available from 19 July 1999 to 23 November 2009, and is widely used in CAGW observational studies^29, 37, 46^.

By vector identity, vertically integrated horizontal moisture flux can be written as:

3 $$\frac{1}{g}{\int }_{70000\,Pa}^{50000\,Pa}q{{\boldsymbol{V}}}_{{h}}{dp}\,{\rm{and}}\,{V}_{h}=u{\boldsymbol{i}}+v{\boldsymbol{j}}$$

where *q* is specific humidity (kg kg^−1^), *u* and *v* represent the standard horizontal wind components (m s^−1^), *p* is air pressure (Pa), and *g* is gravitational acceleration (m s^−2^). Therefore *q* ***V*** has the units of kg kg^−1^ m s^−1^. Since the units of *dp/g*, Pa/(m s^−2^) can equally convert to kg m^−2^, the term ‘mass weighted’ is used for vertical integration. The vertically-integrated moisture flux is in units of kg m^−1^ s^−1^. To convert to a column of water, we assume a constant water density of 10^3^ kg m^−3^, so the units of the vertically integrated moisture flux can be written as mm m s^−1^.

### Data Availability

The datasets generated during and/or analysed during the current study are available in the University Corporation for Atmospheric Research (UCAR) repository [https://rda.ucar.edu/] and National Climatic Data Center (NCDC) repository [https://www.ncdc.noaa.gov/].

## Electronic supplementary material

Supplementary Information
