# Supplementary material for: Intrabasin Variability of East Pacific Tropical Cyclones During ENSO Regulated by Central American Gap Winds
Source: Sci Rep. 2017 May 10;7:1658. doi: 10.1038/s41598-017-01962-3 (PMC5431948; doi:10.1038/s41598-017-01962-3)
Supplement: Supplementary file 1 — Supplementary Information [file 41598_2017_1962_MOESM1_ESM.pdf]

Intrabasin Variability of East Pacific Tropical Cyclones During ENSO Regulated by Central American Gap Winds

Dan Fu[1,2], Ping Chang[1,2,4*], and Christina M. Patricola[3,4]

1 Physical Oceanography Laboratory/CIMST, Ocean University of China and Qingdao National Laboratory for Marine Science and Technology, Qingdao 266100, China.
2 Department of Oceanography, Texas A&M University, College Station, Texas 77843, USA.
3 Climate and Ecosystem Sciences Division, Lawrence Berkeley National Laboratory, Berkeley, California, 94720, USA.
4 Department of Atmospheric Sciences, Texas A&M University, College Station, Texas 77843, USA.
* E-mail: ping@tamu.edu

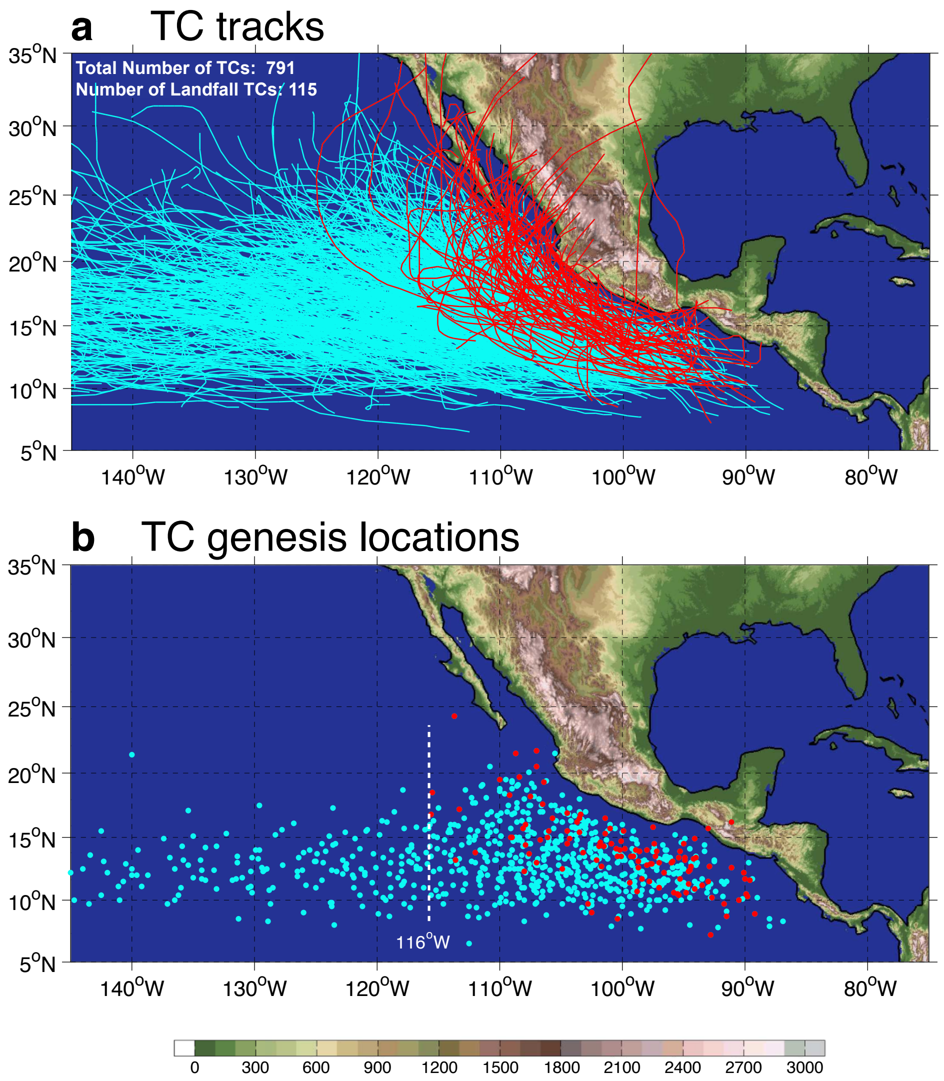

Supplementary Figure S1 | ENP TCs climatology map. Distributions of observed (**a**) TC tracks and (**b**) TC genesis locations over the ENP during 1970 to 2015 hurricane seasons. Red curves and dots indicate TCs that made landfall onto the Pacific coast of Central America and Mexico. Land topography (unit: m) is shaded green-brown. The maps were generated using M_Map V1.4 package for Matlab (http://www.eos.ubc.ca/~rich/map.html).

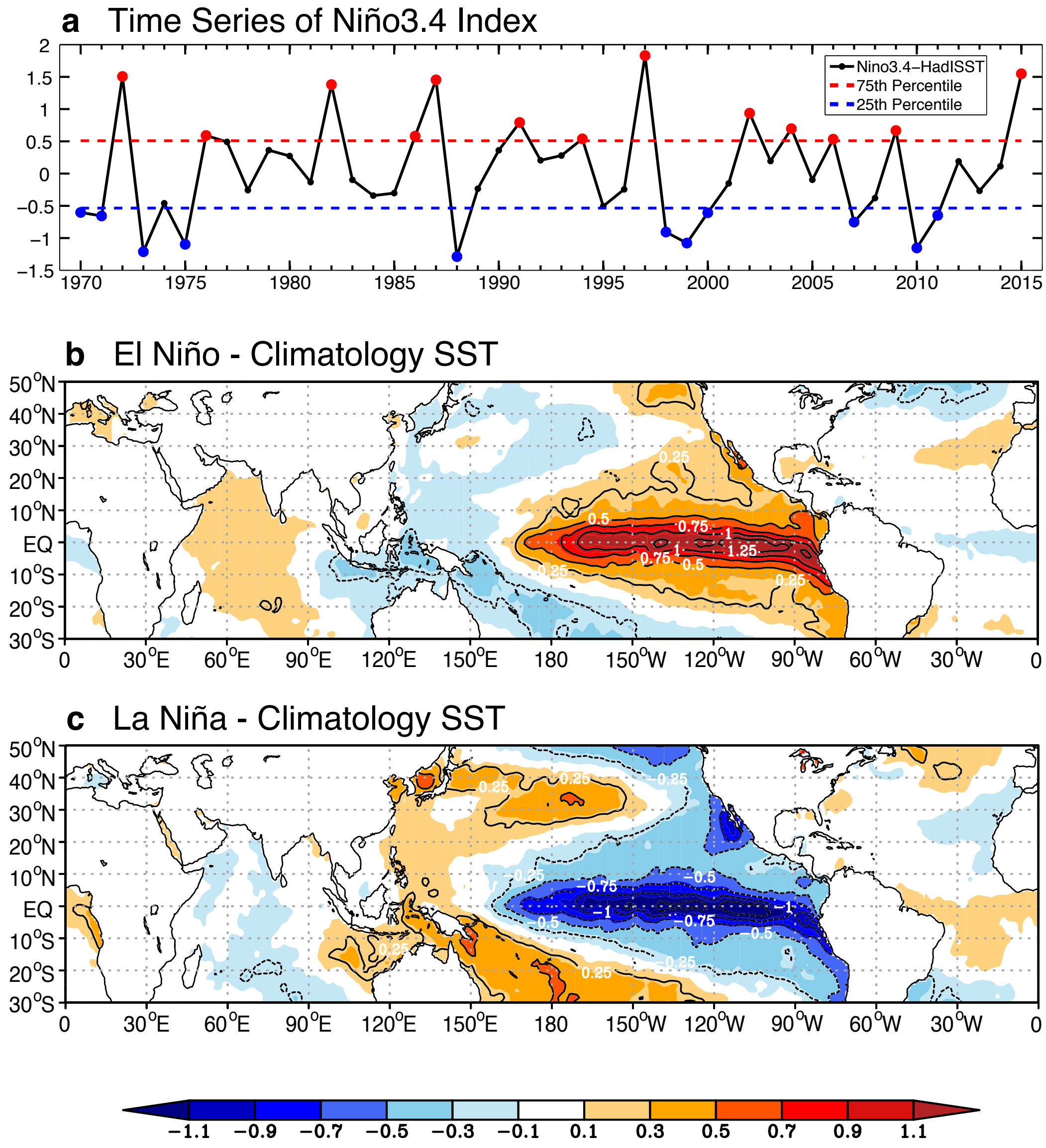

Supplementary Figure S2 | ENSO classification and corresponding SST response. (**a**) Time series of the hurricane season (June -November) mean Niño3.4 index (black curve with dots), with the 75th (red dash) and the 25th percentile (blue dash) values. El Niño and La Niña events are indicated in red dots and blue dots respectively. Difference in hurricane season mean SST from the (**b**) El Niño hurricane seasons (**c**) La Niña hurricane seasons composite minus climatology (contour interval is 0.25 °C). The maps were generated using GrADS V2.1.0 (http://cola.gmu.edu/grads/).

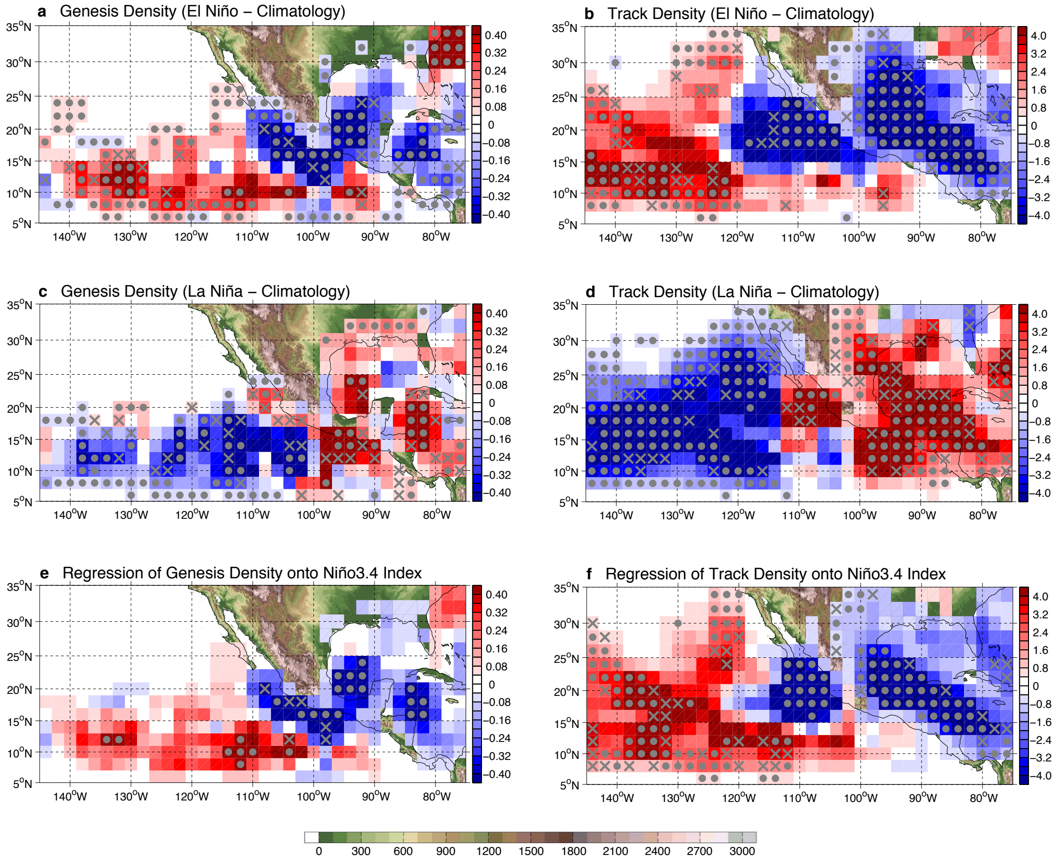

Supplementary Figure S3 | ENP TC variability during El Niño and La Niña. The El Niño hurricane seasons composite minus climatology difference in (**a**) TC genesis density anomaly (unit: TCs per 10 seasons), and (**b**) TC track density anomaly (unit: TCs per 10 seasons) similar to Fig. 1(**a**) and (**b**), except using a bootstrap technique to determine statistical confidence level. (**c**), (**d**) are similar, but for the La Niña hurricane seasons composite minus climatology. Regression of (**e**) TC genesis density and (**f**) TC track density onto the hurricane season averaged Niño3.4 index. Gray dots (crosses) denote statistical confidence at the 95% (90%) level based on the two-sample t-test. Land topography (unit: m) is shaded green-brown. The maps were generated using M_Map V1.4 package for Matlab (http://www.eos.ubc.ca/~rich/map.html).

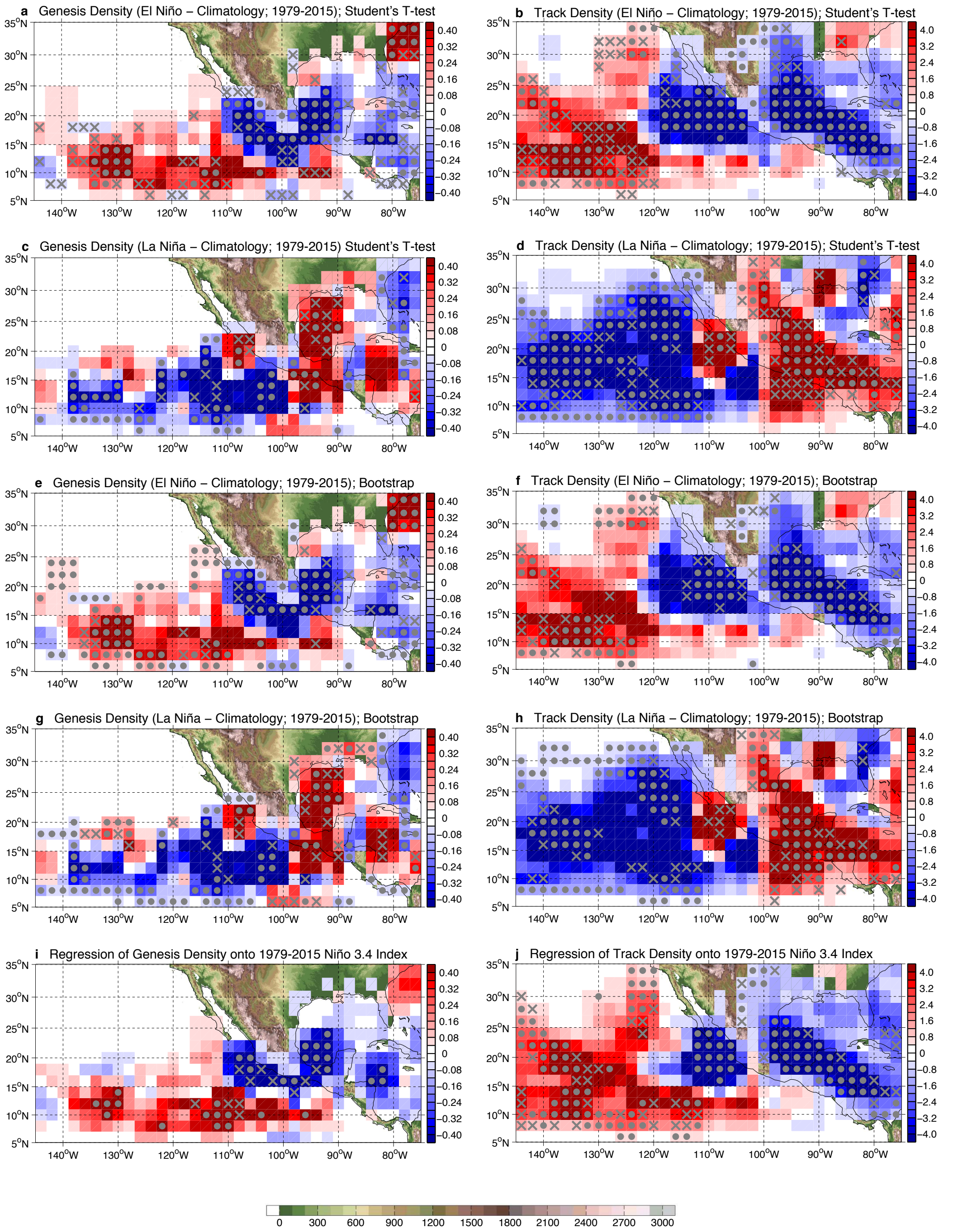

Supplementary Figure S4 | ENP TC variability during El Niño and La Niña (shorter time period). The composite of El Niño hurricane seasons during 1979-2015 minus 37-year (1979-2015) climatology difference in (**a**) TC genesis density (unit: TCs per 10 seasons), and (**b**) TC track density (unit: TCs per 10 seasons), applied by the two-sample t-test to determine statistical confidence level. (**c**), (**d**) are similar, but for the La Niña hurricane seasons composite minus 37-year climatology. (**e**), **(f)**, (**g**) and (**h**) are same as (**a**), (**b**) ,(**c**), and (**d**) but using bootstrap technique to determine statistical confidence level. Regression of (**i**) TC genesis density and (**j**) TC track density onto the hurricane season averaged Niño3.4 index

during 1979-2015. Gray dots (crosses) denote statistical confidence at the 95% (90%) level. Land topography (unit: m) is shaded green-brown. The maps were generated using M_Map V1.4 package for Matlab (http://www.eos.ubc.ca/~rich/map.html).

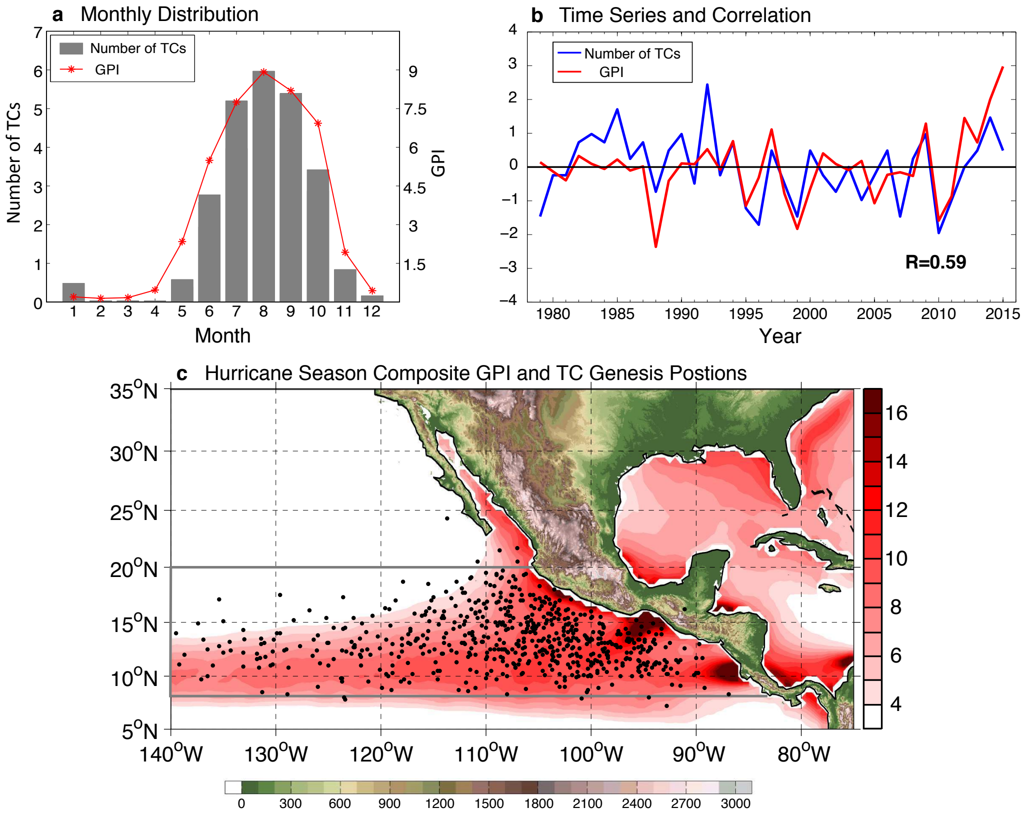

Supplementary Figure S5 | Relationship between genesis potential index and ENP TCs. (**a**) Seasonal cycle of genesis potential index (GPI, unitless, red curve with dots) averaged over the ENP TC main development region (MDR; 8°N-20°N, 85°W-American coast, see gray box in panel (**c**)) and monthly averaged number of ENP TCs (gray barb) from January to December. (**b**) Normalized time series of hurricane season mean GPI averaged within MDR (red curve) and number of ENP TCs (blue curve). (**c**) Climatological hurricane season mean GPI (shading) and all of TC genesis locations (black dots) during 1979-2015 hurricane seasons. Land topography (unit: m) is shaded green-brown. The maps were generated using M_Map V1.4 package for Matlab (http://www.eos.ubc.ca/~rich/map.html).

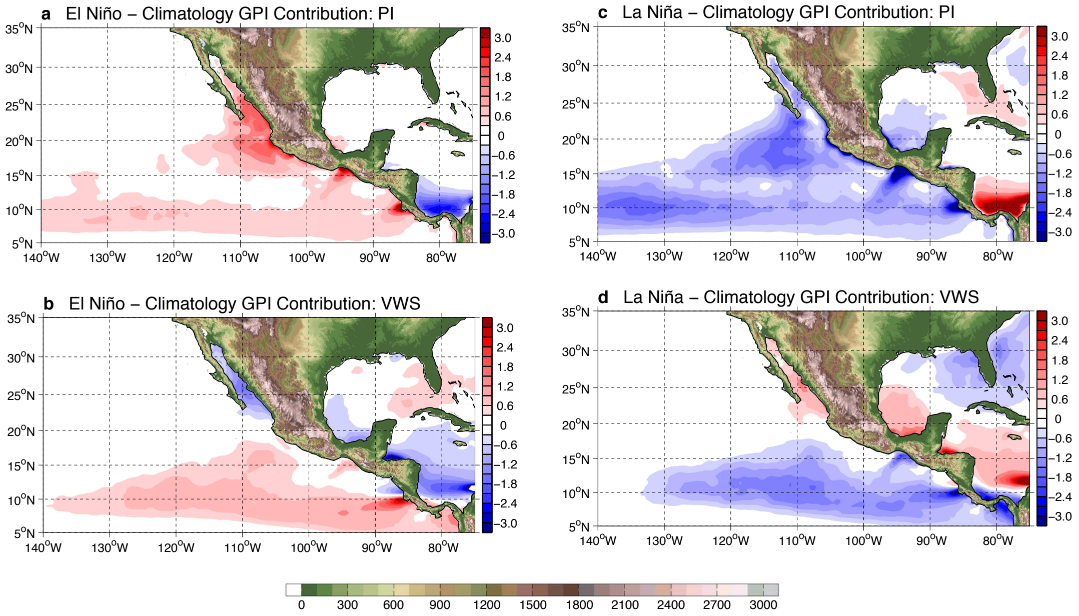

Supplementary Figure S6 | Impact of El Niño and La Niña on environmental favorability for TC genesis. The El Niño hurricane seasons composite minus climatology difference in GPI (unitless) calculated by varying just (**a**) potential intensity (PI[41-43]), and (**b**) vertical wind shear, while setting the other three terms to values of the climatology. (**c**), (**d**) are similar, but for the La Niña hurricane seasons composite minus climatology. Positive indicates environmental conditions are more favorable for TC genesis, and vice versa. Land topography (unit: m) is shaded green-brown. The maps were generated using M_Map V1.4 package for Matlab (http://www.eos.ubc.ca/~rich/map.html).

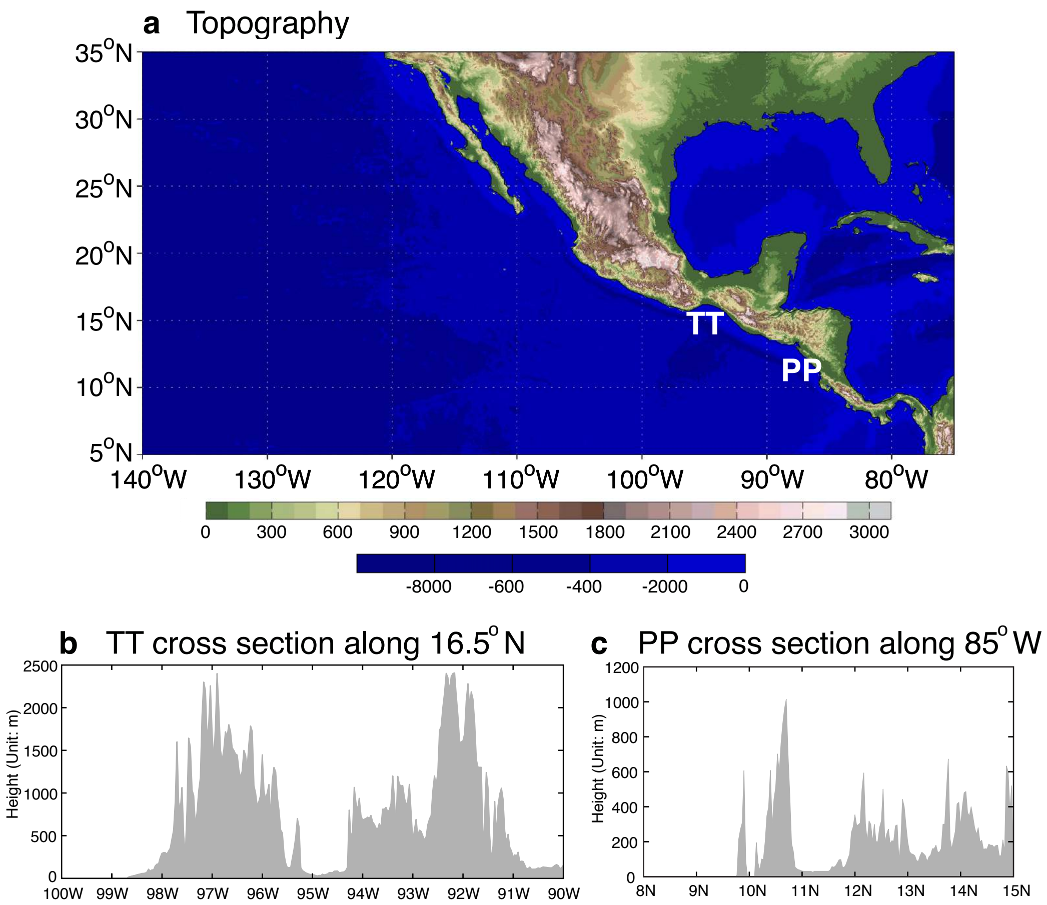

Supplementary Figure S7 | Topography of Central America, Mexico and ENP. (**a**) Land topography (unit: m, top color bar) and ocean depth (unit: m, bottom color bar). Gaps at Tehuantepec, and Papagayo are marked as TT, and PP respectively. Cross sections of surface elevation at (**b**) TT along 16.5°N and (**c**) PP along 85°W. The maps were generated using M_Map V1.4 package for Matlab (http://www.eos.ubc.ca/~rich/map.html).

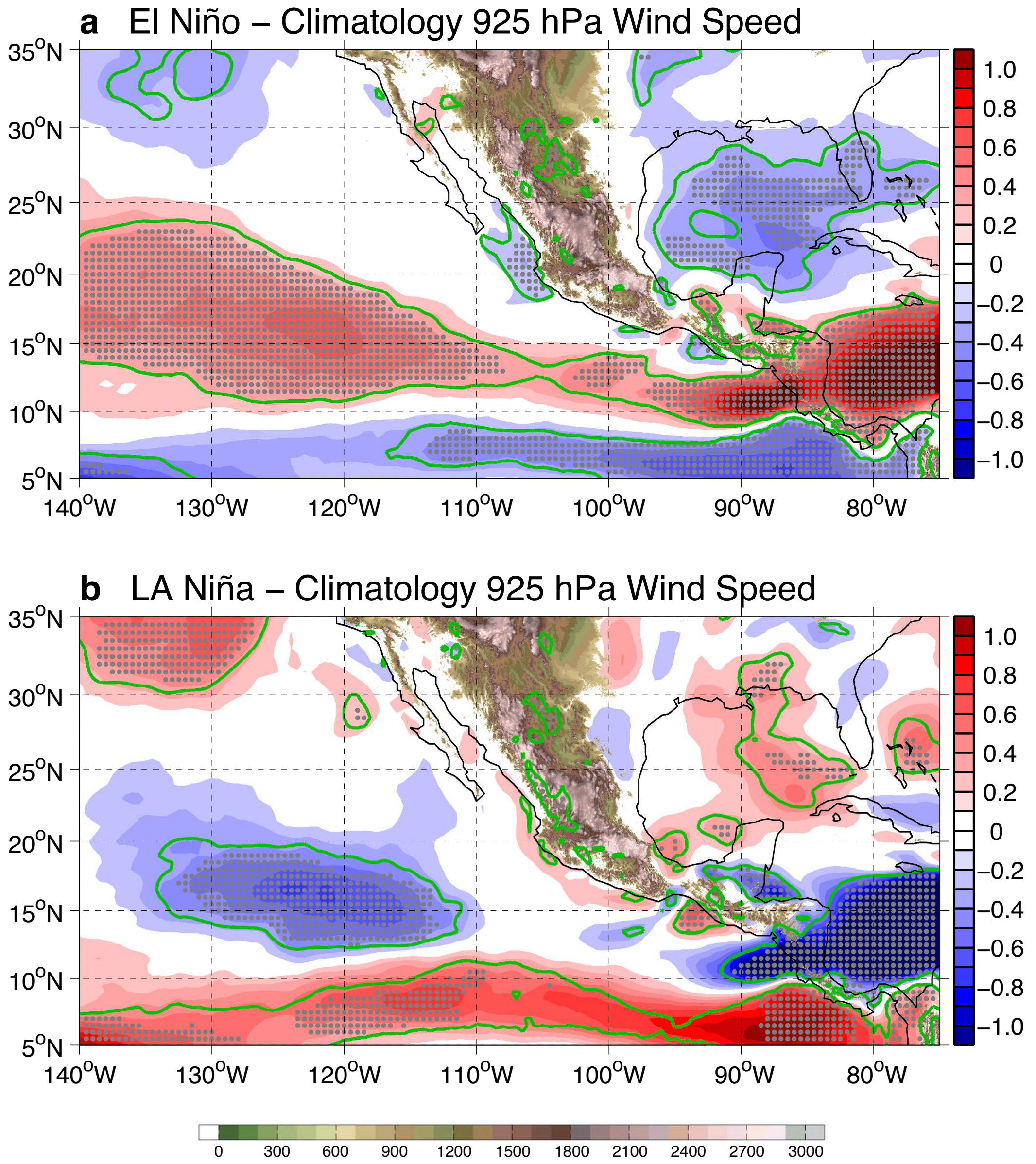

Supplementary Figure S8 | Impact of El Niño and La Niña on low-level wind speed. (**a**) The El Niño and (**b**) the La Niña hurricane seasons composite minus climatology difference in mean scalar wind speed at 925 hPa (unit: m s^{-1}). Gray dots (green contours) highlight the statistical confidence at the 95% (90%) level based on the two-sample t-test. Land topography (unit: m) at atmospheric pressure level is shaded green-brown. The maps were generated using M_Map V1.4 package for Matlab (http://www.eos.ubc.ca/~rich/map.html).
